# Survey of Veterinarians Using a Novel Physical Compression Squeeze Procedure in the Management of Neonatal Maladjustment Syndrome in Foals

**DOI:** 10.3390/ani7090069

**Published:** 2017-09-05

**Authors:** Monica Aleman, Kalie M. Weich, John E. Madigan

**Affiliations:** Department of Medicine and Epidemiology, School of Veterinary Medicine, University of California, Davis, CA 95616, USA; kmweich@ucdavis.edu (K.M.W.); jemadigan@ucdavis.edu (J.E.M.)

**Keywords:** equine, birth, consciousness, hypoxia, neurosteroids, perinatal, survey

## Abstract

**Simple Summary:**

Neonatal foals must pass key milestones for survival such as standing and sucking from the mare shortly after birth. A condition known as neonatal maladjustment syndrome (NMS), or “dummy foal syndrome”, is characterized by failure to stand, suck, and follow their mare, putting them at risk of malnourishment, infection, and death. NMS had been presumed to be exclusively caused by low oxygen in the foal during the perinatal period. More recently, however, our group demonstrated the presence of neuroactive steroids in foals that exhibited the altered behavior and consciousness characteristic of the disorder. It has been hypothesized that signaling the transition from the in utero unconscious state to extrauterine consciousness may involve labor-induced physical compression (squeezing). During normal birth, foals experience such physical compression for approximately 20 min during stage-2 labor. Current medical treatments for NMS are symptomatic and supportive, which may require 2–7 days of veterinary care. Anecdotal evidence demonstrated that a novel physical compression (squeeze method) that applies 20 min of sustained pressure to the chest of neonatal foals exhibiting this syndrome might rapidly hasten recovery. This survey compares reported outcomes of medical therapy alone to this squeeze procedure with or without medical therapy. The results revealed some foals that received the squeeze procedure recovered faster than those that received medical therapy only.

**Abstract:**

Horses are a precocious species that must accomplish several milestones that are critical to survival in the immediate post-birth period for their survival. One essential milestone is the successful transition from the intrauterine unconsciousness to an extrauterine state of consciousness or awareness. This transition involves a complex withdrawal of consciousness inhibitors and an increase in neuroactivating factors that support awareness. This process involves neuroactive hormones as well as inputs related to factors such as cold, visual, olfactory, and auditory stimuli. One factor not previously considered in this birth transition is a yet unreported direct neural reflex response to labor-induced physical compression of the fetus in the birth canal (squeezing). Neonatal maladjustment syndrome (NMS) is a disorder of the newborn foal characterized by altered behavior, low affinity for the mare, poor awareness of the environment, failure to bond to the mother, abnormal sucking, and other neurologically-based abnormalities. This syndrome has been associated with altered events during birth, and was believed to be caused exclusively by hypoxia and ischemia. However, recent findings revealed an association of the NMS syndrome with the persistence of high concentrations of in utero neuromodulating hormones (neurosteroids) in the postnatal period. Anecdotal evidence demonstrated that a novel physical compression (squeeze) method that applies 20 min of sustained pressure to the thorax of some neonatal foals with this syndrome might rapidly hasten recovery. This survey provides information about outcomes and time frames to recovery comparing neonatal foals that were given this squeeze treatment to foals treated with routine medical therapy alone. Results revealed that the squeeze procedure, when applied for 20 min, resulted in a faster full recovery of some foals diagnosed with NMS. The adjunctive use of a non-invasive squeeze method may improve animal welfare by hastening recovery and foal–mare interactions that minimize health problems. This would also avoid or reduce costs arising from hospitalization associated with veterinary and nursing care that sometimes leads owners to elect for euthanasia.

## 1. Introduction

Horses, as a precocious species, are born with a more functional developed brain than altricial species as an evolutionary mechanism for survival in the wild as a prey animal. These evolutionary differences among species are notable in the neonatal period [1]. The transition from in utero unconsciousness and fetal physiology to extrauterine consciousness and activity is critical for survival. Maintenance of the in utero unconscious state is due to a combination of factors. These factors include the neuroinhibitory effects of high circulating and cerebral concentrations of adenosine, progesterone, and its related neuroactive steroids—allopregnanolone and pregnanolone—as well as prostaglandin D2 and a placental neuroinhibitory peptide, all acting together with warmth, buoyancy, and cushioned tactile stimulation, to produce in utero somnolence [2,3,4,5,6]. The fetal foal, as with other precocial mammalian fetuses, remains in a sleep-like state of unconsciousness as brain development progresses during the final 75% of pregnancy. This acts to reduce movement, protecting the mare from the actions of the long limbs of the foal, which can weigh 80–120 lb (37.2–54.4 kg) at birth [6]. Additionally, the placenta and in utero environment achieve respiratory gas exchange independently of fetal breathing, nutrient supply without food ingestion and gut involvement, disposal of metabolic waste products without major involvement of the fetal kidneys and liver, basal rates of thermoregulation, and other functions. All of these must rapidly transition to autonomous activity if the newborn is to survive birth. The mare delivers the newborn over 20–40 min of active labor. During the first few minutes and hours of extrauterine life, foals must achieve these critical milestones, as well as possess the ability to stand up, ambulate, and suck from the mare. Normal neonatal foals bond closely to the mare and have coordinated locomotion compared to maladjusted foals [7]. Any impairment or delay in the achievement of these milestones could result in significant problems of welfare related to inadequate colostrum and milk intake giving rise to energy depletion and weakness, struggling to rise, hypothermia, failure of passive immunoglobulin transfer, infection, and death.

Neonatal maladjustment syndrome (NMS) is a common disorder in neonatal foals with an estimated incidence of 1–2% [8,9]. NMS is a clinical diagnosis based on behavioral observations of reduced awareness of the environment, failure to find the udder and suck, lack of affinity for the mare, wandering, and, in more severe cases, alterations in processes of thermoregulation, reduced intestinal motility, and compromised organ function [9]. The disorder has been referred to as neonatal encephalopathy, perinatal asphyxia, hypoxic-ischemic encephalopathy, and dummy foal syndrome due to the presumption that the condition is exclusively related to hypoxia and loss of cerebral cortical function and death during the perinatal period [9,10,11]. Recent studies found that some foals with NMS have persistence of the neurosteroids progesterone, pregnenolone, androstenedione, dehydroepiandrosterone, and epitestosterone, which are neuromodulators and may possibly be associated with alterations in the state of consciousness [12]. Neuroinhibitory steroids are believed to be partially responsible for keeping the foal in a sleep-like reduced state of consciousness while in utero. The potential neuromodulatory effects in neonatal foals of a neurosteroid known to be sedative or anesthetic in other species was demonstrated by the IV infusion of allopregnanolone to healthy neonatal foals [13,14].

The neonatal foal is very sensitive to squeeze pressure. When manually held tightly, a foal will collapse and become unconscious, a phenomenon described for years as the flopping reaction. Experimentally, when a rope compression system similar to that used in bovine casting was applied to healthy neonatal foals, it produced a state of somnolence and immobility recently termed “squeeze induced somnolence” [15]. Normal healthy neonatal foals developed rapid onset of recumbency, somnolence, and slow wave sleep when subjected to a mid-body compression squeeze procedure using a rope, rapidly regaining alertness when the pressure was removed [15]. Hormonal changes occurred after 20 min of squeeze pressure with an increase in adrenocorticotropic hormone (ACTH), dehydroepiandrosterone sulfate, and androstenedione [15]. The evolutionary value of such immobilization when squeezed might be related to potential benefits of reducing movement of the foal while passing through the narrow birth canal. Since publication of the squeeze method in 2012 [15], anecdotal reports emerged of foals diagnosed with NMS being subjected to the squeeze pressure for 20 min, waking up, and having resolution of NMS clinical signs [16]. The purpose of this paper is to report the results of a survey of veterinarians and farm managers treating NMS foals with the addition of the squeeze method in comparison to those using conventional therapies for NMS. The goal of the brief survey was to determine if the application of this squeeze procedure in neonatal foals with NMS aided the recovery by speeding their attainment of a normal state of consciousness and unassisted sucking from the mare. Additionally, the possible mechanisms involved in the distinctive responses to application and withdrawal of the thoracic squeeze in foals are considered.

## 2. Materials and Methods

The Equine and Comparative Neurology Research Group at the School of Veterinary Medicine, University of California, Davis, invited veterinarians, veterinary technicians, and farm managers to participate in an electronic survey. A request to participate was sent to equine veterinary list servers, referring veterinarians listed in the Veterinary Medicine Teaching Hospital database, notices on web sites listing educational resources, and veterinary meetings. The intent of the survey was to collect observational data from foals with NMS treated with medical therapy and a novel squeeze method. The survey was made available to the target audience from September 2015 to April 2016 via Survey Monkey. Information regarding the step-by-step application of a soft rope squeeze procedure to foals has been provided (Supplementary file). The mild pressure was applied for 20 min using a soft rope looped around the thorax (www.equineneonatalmanual.com/foalsqueezing). The survey reached beyond the United States to include colleagues worldwide.

### 2.1. Electronic Survey

Each participant was informed in writing at the beginning of the survey of the anonymous nature of the survey and was notified of an option to provide contact information for further discussion or participation in future clinical trials. The short survey was designed to improve returns, and to focus on the research question: “does the use of the squeeze procedure modify recovery time from NMS compared to conventional therapies?”

Participants were prompted to answer questions regarding the foaling season during 2015 and the first months of 2016. The first three questions categorized the professions of the participants, practice geographical region, and the number of foals diagnosed with NMS during the stipulated period. Question 4 sought information about medical treatments used such as intravenous fluids, medications, antimicrobials, and others. The next nine questions focused on time for NMS foals to respond to medical treatment only (non-squeezed group) versus the squeeze procedure (squeezed group). A positive response was defined as the foal displaying normal behavior with the mare, and mentation, which produced normal unassisted sucking from the mare. The mentation status of foals before treatment in both groups was reported in four broad categories: normal mentation (bright and alert), obtunded (quiet but responsive to stimuli), stuporous (responsive only to markedly painful stimuli), or comatose (no response to any stimuli). Sucking was categorized as normal when the foal drank from the mare without assistance. Abnormal sucking was defined as being inconsistent and ineffectual including dysphagia (a cough while sucking, milk leaking from mouth or nostrils), or absence of a sucking reflex. Behavior, mentation, and sucking were recorded before and after treatment. The response to treatment for both groups was recorded using broadly defined time periods: those that responded positively in less than 1 h, within 1 h to less than 24 h, 24 to 72 h, and more than 72 h post-treatment.

Written and filmed descriptions of the behaviors of normal and squeezed neonatal foals, also of a NMS foal before and after successful squeezing, were provided to all participants as an integral part of the survey (Manual of Neonatal Equine Medicine—Foal Squeezing with “Madigan Foal Squeeze”: (www.equineneonatalmanual.com/foalsqueezing); YouTube video. Australian foal 2012: (https://www.youtube.com/watch?v=uZ9KpOSN6iU). The distinctive behavioral differences for each category of mentation assessment and the response of the foal as normal was based on unassisted sucking from the mare and was easy to identify.

### 2.2. Data Processing and Statistical Analysis

Survey participants entered responses directly into the study’s account on the Survey Monkey web site. Survey results were downloaded to Microsoft Excel (version 14.2.5) once the survey period was completed. The respondent number was transferred to the Excel worksheet, and each foal was given an identification code. Data was coded and recorded in an Excel worksheet.

Descriptive statistics were calculated using Microsoft Excel. Survey results were summarized by profession, region, number of foals diagnosed, medical treatment choices, number of times squeezed, and adverse effects. Foals were assigned to four treatment groups based on what type of therapy was provided: medical treatment only, squeeze treatment only, medical treatment prior to squeeze, and medical treatment after squeeze.

Logistic regression with a binary outcome was used to compare the medical treatment only group (reference group) with the whole of the squeeze group (all three groups of squeezed foals), as well as the three separate squeezed treatment groups for the response time to normal as determined by ambulation, behavior, and sucking. This generated odds ratios at 95% confidence intervals for recovery time after treatment in the categories of: less than 1 h, within 24 h, and within 72 h. Ordered logistic regression was used to compare the reference group to the squeezed group regarding the recovery scale scored from 1 to 5. A score of 1 represented recovery in less than 1 h; a score of 2, recovery between 1 and 24 h; a score of 3, recovery between 24 and 72 h; a score of 4, recovery in more than 72 h; and score of 5 represented no recovery. Ordered logistic regression was also used to compare all four treatment groups on the recovery scale. The effect of the number of squeezes and overall health status were also examined for effects on recovery time in the squeeze groups. All statistical analyses were performed with STATA/IC version 14.1. A value of *p* < 0.05 was considered statistically significant.

## 3. Results

There were 51 respondents to the survey. These included veterinarians (N = 44/51, 86.6%), and non-veterinarians with experience in foaling at horse farms (N = 5/51, 9.8%), and veterinary technicians (N = 2/51, 3.9%). Eighty-six percent of the respondents were from the United States. The remaining 14% were from Africa, Australia, Canada, and Europe. The number of foals diagnosed with NMS per respondent ranged from one to more than eight. Information was available for 195 foals. All foals exhibited abnormal behavior, mentation, and/or sucking, typical of NMS. Foals were grouped into non-squeezed (N = 108) and squeezed (N = 87) groups. Signalment was not available for all foals. However, all foals included in this study were treated within 24 h of birth. All foals were of Thoroughbred and Quarter Horse breeds. Sex was not reported for all foals.

Respondents reported using a variety of medical treatments in 108 foals. Medical treatment included tube or bottle feeding (89.6%), plasma administration (83.3%), intravenous fluids (81.3%), antimicrobials (79.2%), dextrose administration (52.1%), dimethylsulfoxide (43.8%), vitamin E (43.8%), intranasal oxygen (39.6%), diazepam (27.1%), corticosteroids (25%), mannitol (20.8%), and allopurinol (10.4%), and other miscellaneous treatments including vitamin C, thiamine, caffeine, naloxone, and hyperbaric oxygen therapy (29.2%). Of the 87 foals in the squeezed group: 21 (24.1%) did not receive any additional treatment, and 42 (48.8%) and 24 (27.6%) received medical treatment prior to and after the squeeze procedure, respectively. Medical treatment for the squeezed group was not specified.

Fifty-eight foals (66.7%) were squeezed once, whereas 23 (26.4%) and 6 (6.9%) were squeezed twice and more than twice, respectively. Time to respond to treatment and odds ratios for recovery of foals in this survey are reported in Table 1 and Table 2. There were statistically significant differences at different time points regarding time to respond positively to treatment between the non-squeezed group and the squeezed group. Foals that received the squeeze procedure with or without medical therapy were 3.7 times more likely to recover faster than foals that did not receive a squeeze (*p* < 0.001, Table 2). Foals that were squeezed had significantly faster and higher recovery rates at different time points. Foals that were squeezed were 15.1 times more likely to recover in less than 1 h than foals that were not squeezed. Foals receiving only squeeze treatment were 17.5 times more likely to recover within the first 24 h than foals treated with only medical treatment. Note that recovery did not occur in 12% of all foals and in 14% of squeezed foals.

The health status determined by physical examination of the squeezed foals before treatment began did not significantly affect the choice of treatment (medical treatment before or after squeeze), the recovery scale, or the number of squeezes. There were no complications with the squeeze procedure in 86 foals, and 1 foal was reported to have transient aspiration of milk after the procedure.

## 4. Discussion

This study showed that foals that were squeezed for 20 min had significantly faster and higher recovery rates at different time points than foals that were not squeezed. Further, foals that received the squeeze procedure only, without medical therapy, were 17.5 times more likely to recover within 24 h than the group receiving only medical therapy. The majority of squeezed foals (N = 59/87, 68%) recovered within 24 h post-squeeze compared to foals that were not squeezed (N = 38/108, 35%). Only 3.4% (N = 3/87) of foals in the squeeze group took more than 72 h to recover versus 14.8% (N = 16/108) foals from the medical group. The overall recovery rate was 86% and 87% for the squeeze and non-squeeze groups, respectively. This is in agreement with the recovery rate of 80% reported previously [9]. However, the squeeze procedure appeared to be useful in decreasing the time to full recovery.

The squeeze procedure can have several advantages including avoiding referral of uncomplicated cases to veterinary intensive care institutions, reduction of costs of nursing care, reduction of time and effort spent by attending care givers and veterinarians, and faster recovery. Further, the option of euthanasia due to financial constraints, lack of personnel or resources to provide adequate nursing and intensive care, and/or perception of poor prognosis due to severity of signs, can potentially be avoided. With regard to lack of complications and evidence of safety, the survey results are similar to findings of experimental use of the squeeze procedure in normal foals [15]. Contraindications to use of the squeeze procedure in foals include factors such as respiratory compromise, neuromuscular disease such as botulism, cardiomyopathies, and broken ribs.

**Figure 1:**
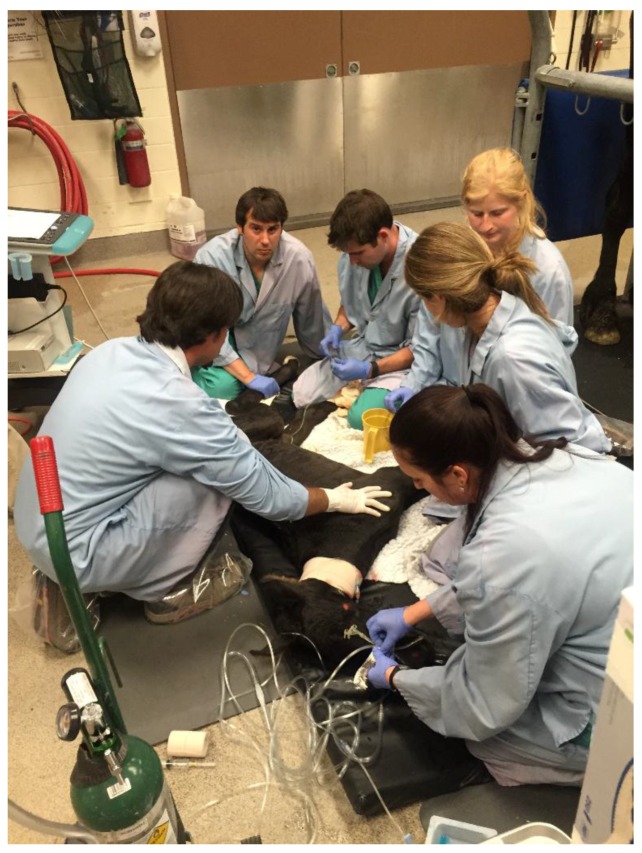
Neonatal foal being received and evaluated in an intensive care referral veterinary hospital. NMS foals may need a controlled environment with minimal pathogens and ideal temperature, often with accommodations for the mare nearby. The mare may need to be milked and the foal tube fed frequently when the foal lacks a suck reflex. Continuous care and observation by trained personnel may be required, including intravenous fluid and medication administration. Critical care facilities have access to laboratory measurements required for direct supportive care. Recumbent foals may require physical therapy and body position support. Methods that can be used in the field setting that result in the foal more rapidly sucking the mare may prevent secondary complication and the need for referral to critical care facilities.

We propose that the pressure of the birth canal during stage-2 labor might play a role in the neonatal transition from neuroinhibition to neuroactivation. NMS has been commonly associated with events that might produce brain hypoxia and ischemia, as well as fetal stress [17]. A condition known as premature placental separation associated with fetal oxygen shortage, and which necessitates rapid assisted delivery of the foals resulting in a substantial reduction of time spent in the birth canal, suggested that one or both of these factors might contribute to such foals having a period of maladjustment [18,19]. In healthy neonatal foals, the concentrations of neurosteroids declined steadily to very low concentrations within 24 h of birth compared to those of foals with NMS [12,20,21]. A persistent elevation of neurosteroids is observed in foals with NMS 24 h post-birth [12]. The concept that NMS in some foals results from a delay in clearance of sedation-producing compounds or a reversion to persistent production of sedative neurosteroids could explain the historical finding of full recovery in 80% of neonatal foals with NMS without residual neurological deficits when provided with nursing and supportive care. Such nursing care is expensive and it is not known how many foals with NMS die in the field when access to such care is not available or affordable to the owner (Figure 1). If hypoxia was the common etiology of the severe neurological signs of NMS, it would be expected to produce some degree of neuronal injury resulting in an early death, and, if not, subsequent long term brain or spinal cord compromise as evidenced in other species following severe hypoxia [12,22].

When considering the induction of immobility and somnolence by the squeeze procedure in newborn foals [15] and the role that thoracic compression might have during uncomplicated successful birth, Mellor offered the following possible explanation which he integrated into his earlier accounts of the mechanisms responsible for the rapid transition from prenatal unconsciousness to postnatal consciousness [1,2,3,4,5,6] (see Mellor’s commentary below).

David Mellor, Animal Welfare Science and Bioethics Centre, Massey University, New Zealand

**Transitions in neuroinhibition and neuroactivation in neurologically mature young at birth, including the potential roles of thoracic compression during labor.**

The following brief account of changes in the balance between inhibition and activation of cerebral cortical function before, during, and after birth is based on published, fully referenced reviews [1,2,3,4,5,6,23] and information provided in the present paper.

During at least the last one-third of pregnancy, cerebral cortical electrical activity indicates the states of fetal unconsciousness, which require less oxygen to sustain than does consciousness. At least nine neuroinhibitory factors unique to life in utero apparently contribute to this; namely, high circulating and/or cerebral concentrations of adenosine, progesterone, allopregnanolone, pregnanolone, prostaglandin D2, and a placental neuroinhibitory peptide, plus warmth, buoyancy and cushioned tactile stimulation. Nevertheless, fetal movements continue to occur until labor begins. These factors play a part in fetal cortical inhibition. However, adenosine—which is released very rapidly during hypoxemia—is particularly capable at switching off cerebral cortical electrical activity, thereby protectively reducing cortical oxygen consumption by at least 95% during marked oxygen shortages. Reoxygenation within about 6 min reverses these effects without neural damage. This has relevance to episodes of transient hypoxemia during labor as well as after severance of the umbilical cord and before the successful onset of breathing.

Neuroinhibition through these factors continues during labor. At the same time, however, two potent hormonal neuroactivators having widely distributed excitatory cerebral effects become increasingly manifest. The first is estradiol-17β, whose fetal plasma concentrations rise throughout labor due to redirection of placental steroidogenesis away from progesterone under the action of the prepartum surge-release of fetal cortisol, which initiates labor. The second is noradrenaline, which is released from the locus coeruleus nucleus giving rise to fibers innervating extensive areas throughout the brain and acting to stimulate arousal and alert vigilance. Potent stimuli to locus coeruleus noradrenaline release include hypoxemia and hypercapnia, strong tactile stimulation of the head and body (squeezing), and injury-induced pain, which may all occur during labor and delivery. These factors presumably prime the brain to facilitate the onset of arousal and consciousness soon after birth, aided by a postnatal withdrawal of the in utero neuroinhibitors.

For the transition to consciousness to occur postnatally, such neuroactivation during labor must not overcome the in utero neuroinhibition until after birth. It is suggested that compression of the thorax during labor may activate a yet undescribed neuroinhibitory reflex that counterbalances the labor-related stimuli that promote neuroactivation via locus coeruleus-noradrenergic pathways. The operation of such an overriding reflex through a different neural pathway is suggested by the rapidity of the distinct behavioral responses to application and withdrawal of the thoracic squeeze procedure [15]. An additional benefit to thoracic squeezing would be the suppression of fetal movements during labor [15]. A similar inhibitory neural reflex has been demonstrated in newly hatched chicks. These chicks rapidly become immobile and exhibit somnolent EEG patterns when returned to the pre-hatched neck-folded posture they had in the egg, effects that are rapidly reversed when the neck is unfolded

**Figure 2:**
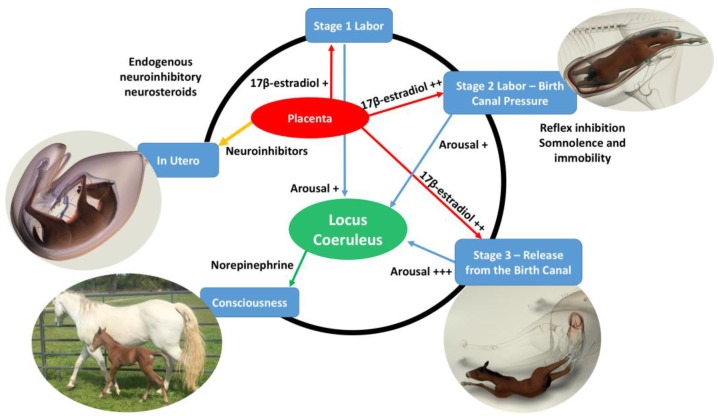
Illustration of the proposed influences associated with the transition of consciousness from intrauterine to extrauterine life. While in utero, the fetal foal remains in a sleep-like state of consciousness, which acts to protect the mare from movements of long limbs of foals weighing 80–120 lbs (37.2–54.4 kg) at birth. This neuroinhibited state is maintained in mammals through high circulating and cerebral concentrations of adenosine, progesterone and its related neuroactive steroids allopregnanolone and pregnanolone, as well as prostaglandin D2 and a placental neuroinhibitory peptide, all acting together with warmth, buoyancy, and cushioned tactile stimulation [2,3,4,5,6]. At the onset of stage-1 labor the fetus comes under the stimulation of 17β-estradiol, which continues through stages 2 and 3 affecting the locus coeruleus. The locus coeruleus produces large amounts of norepinephrine, which causes the transition to consciousness at birth. During stage-2 labor, the arousal stimulation is overridden by thoracic pressure that induces squeeze-induced somnolence and immobility while in the narrow birth canal. When the fetus exits the birth canal, the squeeze pressure is released and full stimulation from the priming of 17β-estradiol occurs. The foal transitions to full consciousness and mobility shortly after delivery, which is essential for survival as a prey animal.

**Figure 3:**
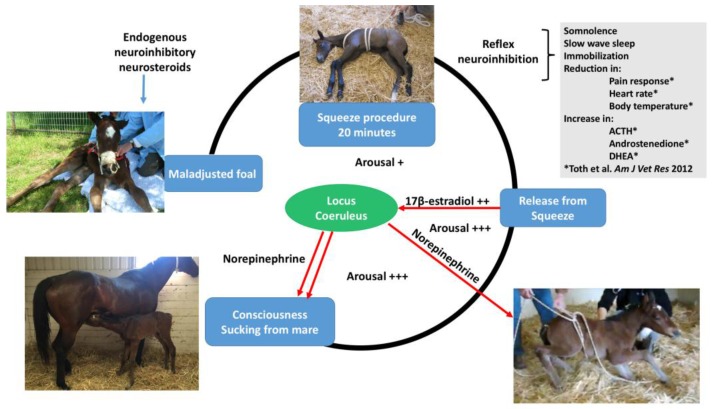
Illustration of the factors associated with the squeeze induced transition of consciousness in a foal with neonatal maladjustment syndrome. A persistent elevation of neurosteroids is observed in foals 24 h post-birth [12]. When the foal is subject to the 20-min squeeze procedure, a reflex neuroinhibition occurs with onset of somnolence; slow wave sleep; immobilization; and reduction in pain, body temperature, and heart rate [15]. Endocrine changes occurring during the squeeze include increased ACTH, androstenedione, and DHEA [15]. Release of the squeeze pressure produces arousal and transition to normal consciousness in some foals with onset of sucking the mare. In other mammals, onset of consciousness is associated with 17β-estradiol stimulation, which affects the locus coeruleus that, in turn, produces large amounts of norepinephrine, creating the transition to consciousness at birth [2,3,4,5,6].

After birth, key neuroinhibitors are withdrawn rapidly. Thus, thoracic compression ceases immediately and elevated adenosine concentrations due to labor-induced hypoxemia decline as oxygenation of all newborn tissues markedly increases with the successful onset of breathing. Likewise, the supply of placental peptide neuroinhibitor and any remaining placentally derived progesterone ceases with severance of the umbilical cord. Withdrawal of inhibitors, including allopregnanolone and pregnanolone, is slower. It is apparent, however, that multifactorial neuroactivation at this time is usually sufficient to counter any persistent but declining neuroinhibition, thus promoting the onset of consciousness or awareness (Figure 2 and Figure 3). Such factors include estradiol-17β and noradrenaline, as well as ambient cold, gravity, unfamiliar contact with hard surfaces and air, changes in the quality of sound, the experience of sight, contact with the mother, and other sensory inputs.

**Figure 4:**
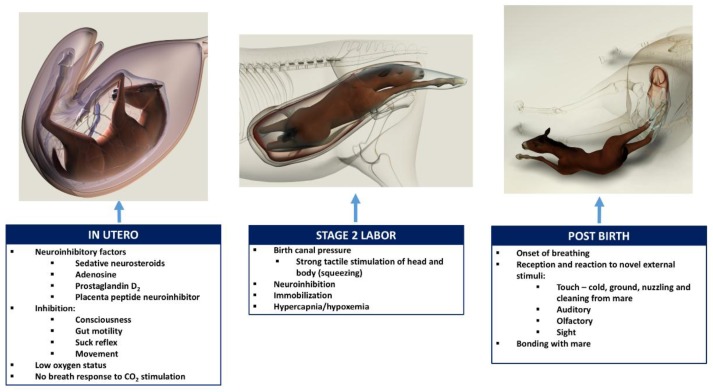
Illustration of the proposed influences associated with states of consciousness, physiological functions, and physical factors affecting the fetal foal in utero, stage 2 labor, and immediately post-birth. In utero, neuroinhibitory factors cause inhibition of movement and consciousness. At this stage, the fetus receives oxygen and nutrition from the placenta, and organs such as the gut and lungs are quiescent. During stage 2 labor, birth canal pressure produces immobility and further neuroinhibition associated with mild hypoxemia and hypercapnia. Immediately upon exiting the birth canal, thoracic squeeze pressure is reduced, releasing neuroinhibition. Physical factors such as skin sensation, olfactory, auditory, and visual stimuli contribute to arousal, ultimately leading to bonding with the mare.

The above account, as well as observations by the present authors [14], suggest that applying the thoracic squeeze procedure to postnatal NMS foals for 20 min would simultaneously elicit two responses mediated by different neural pathways. The first response is reflexive inhibition of physical movement and electrocortical activity leading to immobility and somnolence. The second response is locus coeruleus release of neuroactivating noradrenaline. If so, it is apparent that the former reflex-induced somnolence would dominate the locus coeruleus activation that would continue throughout the duration of the compression. Withdrawal of that inhibition after 20 min of locus coeruleus neuroactivational priming would rapidly free the cerebral cortex to express consciousness (Figure 4) [24].

**Figure 5:**
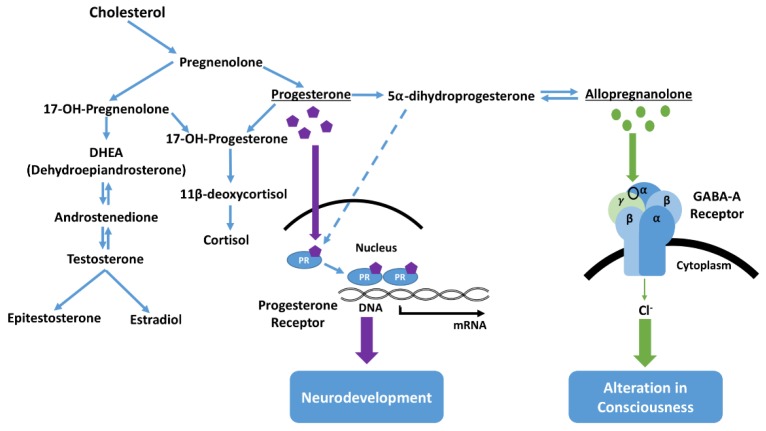
Synthesis and pathways of neurosteroids in the brain [27]. Neurosteroids can have potent effects on both neurodevelopment and consciousness [12]. Progesterone and its related derivatives have been reported to affect gene transcription important for neurodevelopment. Additionally, progesterone and deoxycorticosterone serve as precursors for the endogenous neurosteroid allopregnanolone, which acts on the GABA receptor to rapidly alter consciousness. Levels of neurosteroids in the blood, central nervous system, and spinal fluid are distinct, and may be influenced by levels of precursor steroids shown here. Elevated blood levels of progesterone, pregnenolone, androstenedione, dehydroepiandrosterone, and epitestosterone have been reported in foals with NMS, and are possibly associated with behavioral and conscious state alterations [12].

A complex question remains concerning the postnatal role elevated neuroactive steroid levels may have in the etiology of NMS in foals [13]. Interpretation of associations between behavior and neurosteroid levels are complicated, as levels in plasma might differ from those in the central nervous system (CNS) and cerebrospinal fluid [25]. Steroid precursors that enter the CNS may be involved in the conversion, synthesis, and metabolism of steroids within the specific areas of the brain [25], for example, intravenous infusion of cholesterol produces a state of anesthesia [26]. Neural cells also express enzymes which may catalyze the conversion of progesterone into dihydroprogesterone, which may itself be further converted into other neuroactive steroid metabolites (Figure 5) [27]. The classification of neurosteroids as stimulatory or inhibitory has been largely based on experimental examination of direct effects on receptors such as gamma-aminobutyric acid (GABA) receptors [25]. Pregnenolone, found at high concentrations in foals with NMS, can be converted to progesterone, which can have an inhibitory effect on the CNS. Therefore, it seems reasonable to note the association in ill foals between aberrant behavior and high levels of pregnanes (1200× in some cases), as well as progesterone [13]. One explanation is that these elevated neurosteroids are a persistence of the sedative neurosteroids that keep the fetal foal unconscious, and NMS represents a failure to fully transition consciousness from the in utero state to extrauterine life. Further complexity is added with the finding that receptors in the fetal brain are more sensitive to pregnanes than are those of the adult brain [28]. Whatever the precise roles different chemical neuroinhibitors and neuroactivators might have in NMS, successful use of the thoracic squeeze procedure can apparently reset cerebral cortical function in many affected foals. Locus coeruleus priming for neuroactivation, expressed once the overriding reflex induction of immobility and somnolence is withdrawn at the end of the squeezing, is apparently sufficient to overcome any continuing in utero inhibition to successfully refresh, or reset cortical function to better cope with postnatal life.

In human infants, skin to skin holding of the infant is commonly used and is termed kangaroo mother care or skin-to-skin contact [29,30,31,32,33]. This procedure has been proven to improve premature infant survival and has lasting effects on neurodevelopment [30,31]. Recently, the effect of skin-to-skin contact in infants on the natural postnatal decline in neurosteroids has been reported [33]. Interestingly, infants delivered by caesarean section had less of a decline in three neurosteroids over the first two days of postnatal life than infants of normal vaginal deliveries [33]. Neurosteroids can modulate neurotransmitter receptors in adults, but importantly, during neurodevelopment neurosteroids, have additional functions [34]. Neurosteroids are involved in neuronal modeling. For example, dehydroepiandrosterone (DHEA) and dehydroepiandrosterone sulfate (DHEAS) stimulate axonal growth and have effects on neurotransmitter expression [35]. Touch is the first sense to emerge in utero and is the most strongly developed at birth [1,36]. Touch sensors in the skin are associated with nerve fibers connecting to the CNS. Both elements of the suggested mechanism underlying the changes in consciousness in the NMS foal when squeezed involve touch-stimulated sensory inputs to the CNS [36].

## 5. Limitations

Limitations of this survey study include an inability to ensure equal randomized representation of respondents who had used the squeeze procedure with favorable results and others where the outcomes were unfavorable. Hence, it is possible that more respondents achieving a positive outcome responded and those where no change was noted may not have responded. A second limitation was difficulty in providing training to ensure proper application of the procedure, although links to demonstration videos were provided. Finally, the reliability of reporting the time frames following the use of different therapies was dependent on those completing the survey. An attempt to mitigate this was the use of broad time categories for responses to squeezing, for example, less than 1 h and then >24 h.

## 6. Conclusions

The responses to this survey indicate that a squeeze procedure applied for 20 min resulted in faster full recovery of some foals diagnosed with NMS compared to other foals treated medically only. No side effects were noted by the survey respondents. Animal welfare may be improved by hastening recovery and by foal–mare interactions that minimize health problems. This would also avoid or reduce costs due to hospitalization associated with veterinary and nursing care that sometimes lead owners to elect for euthanasia.

## Figures and Tables

**Table 1:** Response to treatment at different time points post-medical and post-squeeze treatment.

Treatment	<1 h	<24 h	24–72 h	>72 h	Recovery	No Recovery
Medical (N = 108)	4	34	40	16	94	14
%	4	35 ^1^	72 ^2^		87	13
Squeeze (N = 87)	32	27	10	6	75	12
%	37	68 ^1^	79 ^2^		86	14
Total (N = 195)	36	61	50	21	169	26
%	19	50 ^1^	75 ^2^		87	12

^1^ Percentage of foals that recovered by 24 h (includes <1 h); ^2^ Percentage of all foals that recovered by 72 h.

**Table 2:** Odds ratio (OR) and confidence interval (CI) for recovery outcomes. Non-squeezed group versus squeezed group.

Recovery Scale Time Recovery	OR	95% CI	*p* Value
Faster Recovery Scale	3.7	2.13–6.5	<0.001
<1 h	15.1	5.09–49.97	<0.001
<24 h	3.8/3.9	2.13–7.06	<0.001

## Supplementary Materials

The following are available online at www.mdpi.com/2076-2615/7/9/69/s1, supplementary file title: Step-By-Step Written Instructions.
