# Supplementary material for: Survey of Veterinarians Using a Novel Physical Compression Squeeze Procedure in the Management of Neonatal Maladjustment Syndrome in Foals
Source: Animals (Basel). 2017 Sep 5;7(9):69. doi: 10.3390/ani7090069 (PMC5615300; doi:10.3390/ani7090069)

1. SUPPLEMENTARY FILE

Step--- By--- Step Written Instructions

Pre--- squeeze Rope Preparation:

1. Tie a bowline knot and make a fixed loop so that the rope will slide through like a honda on a lariat.
2. How to tie the bowline knot: http://www.animatedknots.com/bowline/

Rope Placement: Starting at the withers area, place rope across the neck and between the front legs and bring end of rope up to withers area (see Step 1 Photo). Thread the end of the rope through the fixed loop and adjust to a snug fit so there is pressure on the chest but no airway obstruction (see Step 2 Photo). Pass the rope over the foal and make a half hitch--- --- --- snug the rope just behind the elbow where a cinch would go on a saddle (see Step 3 Photo). Pass the rope over the foal's ribcage again about 6 inches back from the first half hitch and snug it up (see Step 4 Photo). Have the assistant hold the foal and position yourself behind the foal and put pressure on the rope until the foal begins to lie down (see Step 5 Photo).

Squeezing: Keep the same pressure on the foal when it lies down and same pressure for the 20--- minute duration of the squeeze (see Step 6 Photo). Allow the mare to be standing close viewing the foal. They seem to know the foal is asleep and stand watch. Protect yourself from the foal kicking or moving its head during this time. Some foals seem to move once or twice during the 20 minutes of squeeze. The amount of pressure is just the amount needed to have the foal lie down. When you pull on the rope and the foal begins to lie down, keep that same pressure on for 20 minutes. Using a luggage scale, it should register at about 10--- 20 lbs of pressure.

At the end of the 20--- minute squeeze, release pressure on the rope and allow the foal to stay down or get up as it chooses. Slowly move the rope away from the foal. Do not force it to get up. Let the foal sleep more if it wants. When the foal gets up, allow it to do whatever it wants and observe. Do not attempt to assist nursing or push to the mare.

Step--- By--- Step Instructions with Photos

Step 1: Put the rope over withers and between front legs.

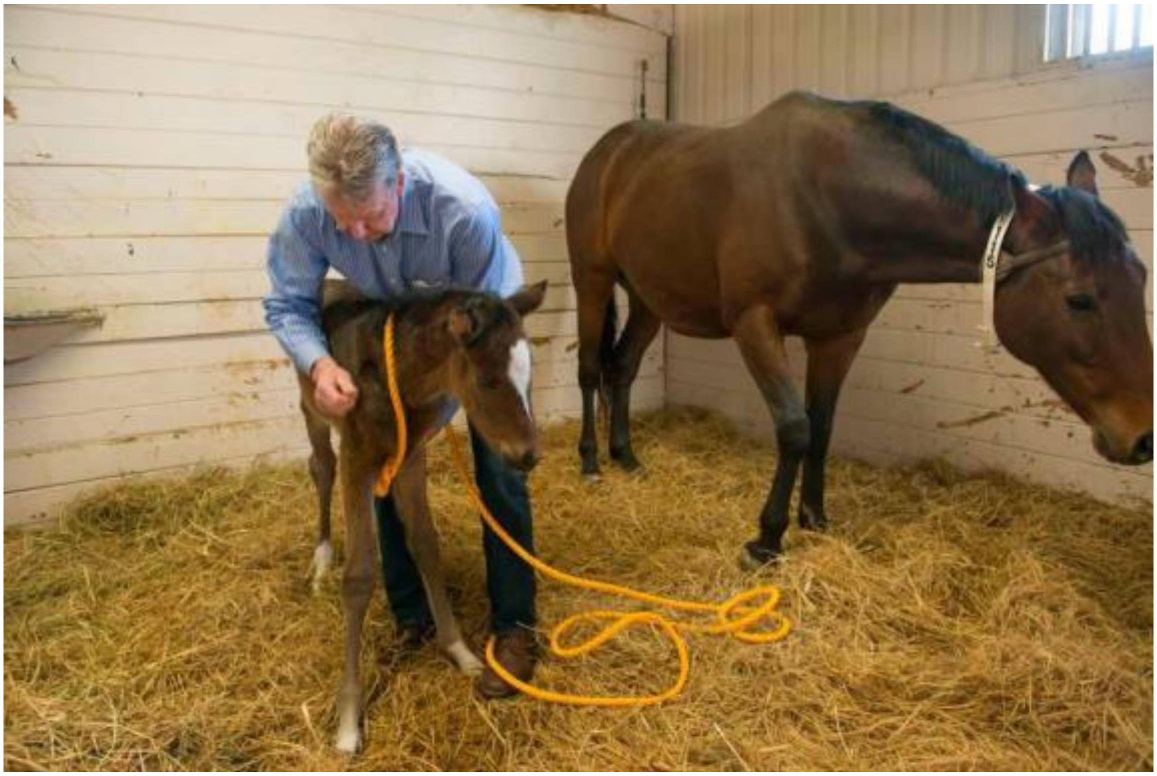

Step 2: Run the rope through the loop at end of the rope and snug rope.

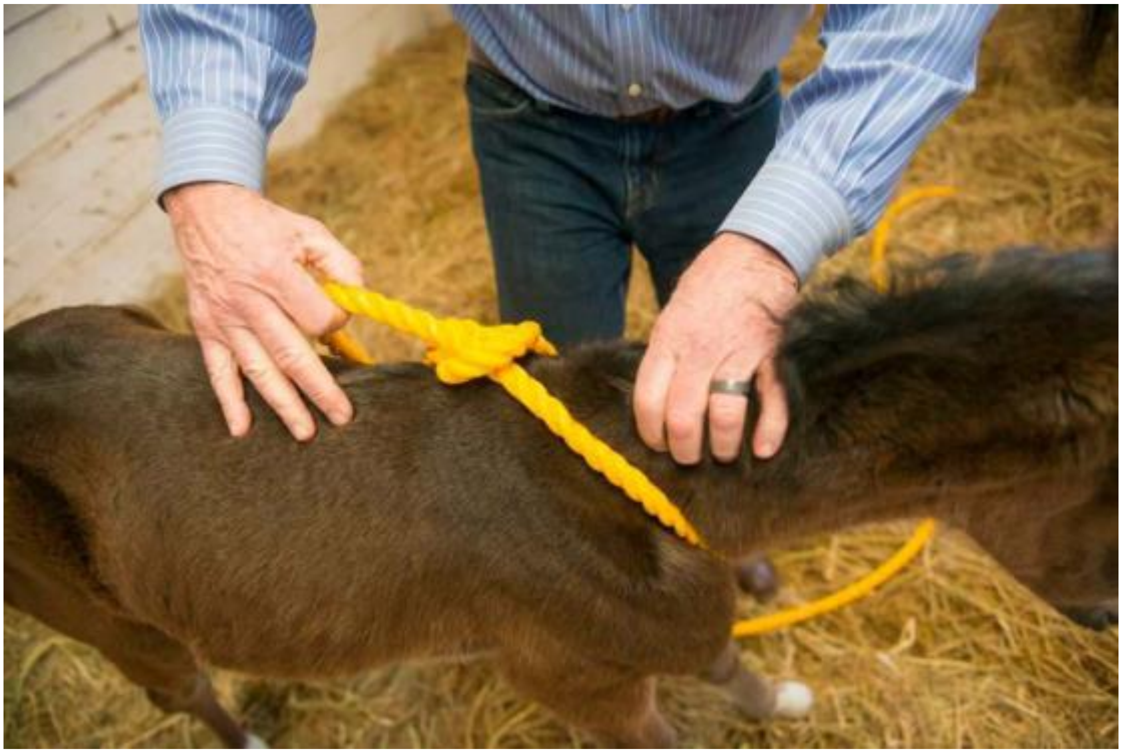

Step 3: Begin by making the first half hitch around the chest, just behind elbow.

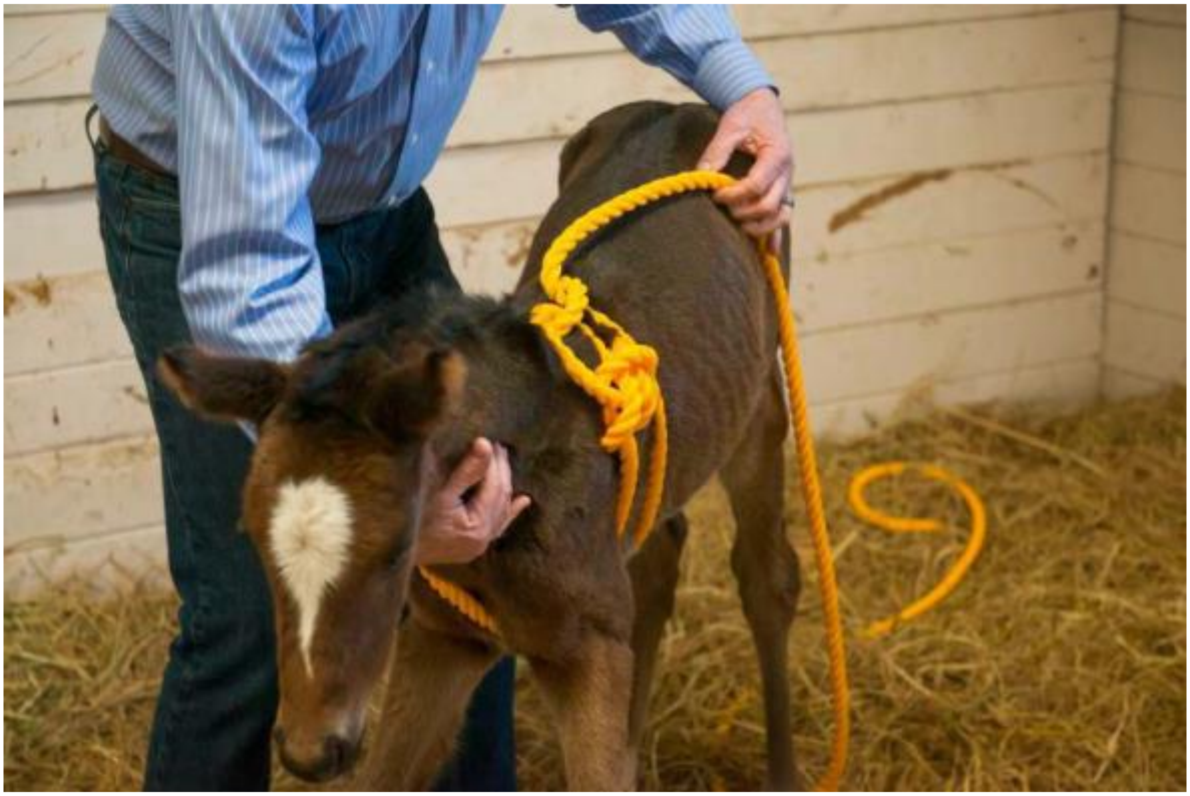

Step 4: Keep the rope snug (photo of first half hitch in place)

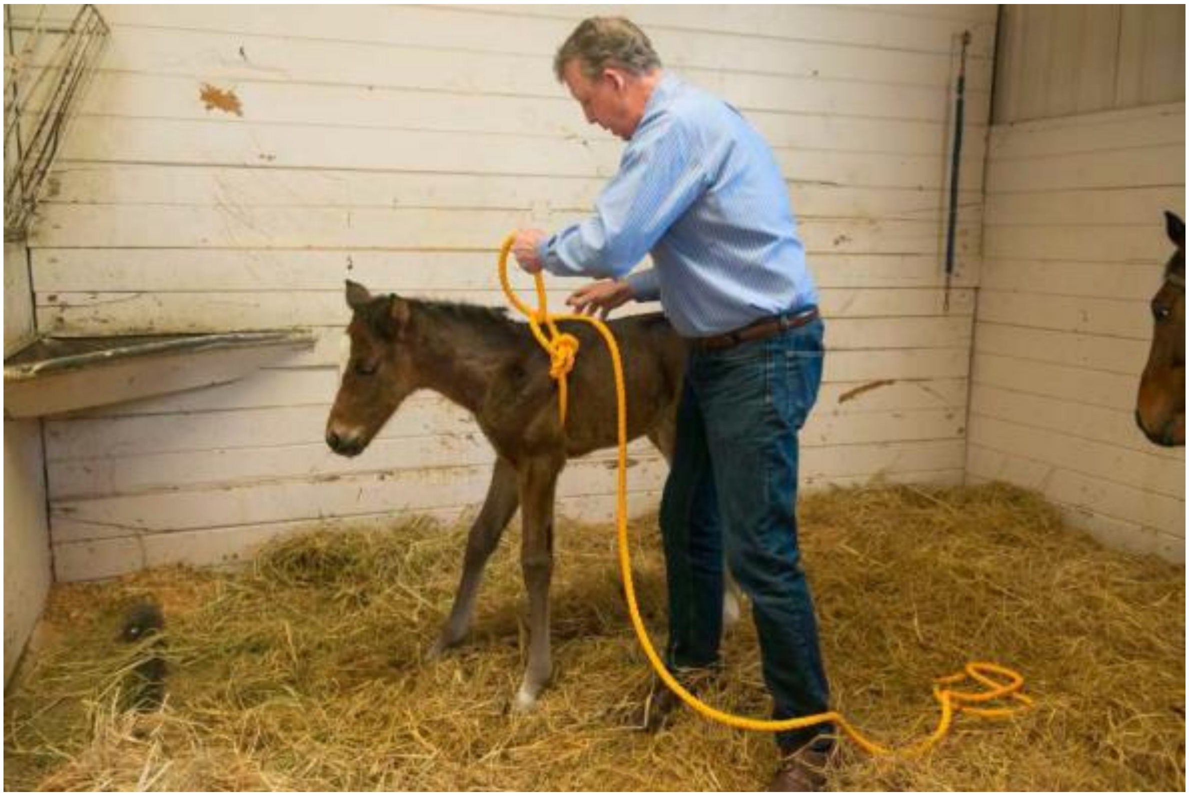

Photos of two half hitches in place

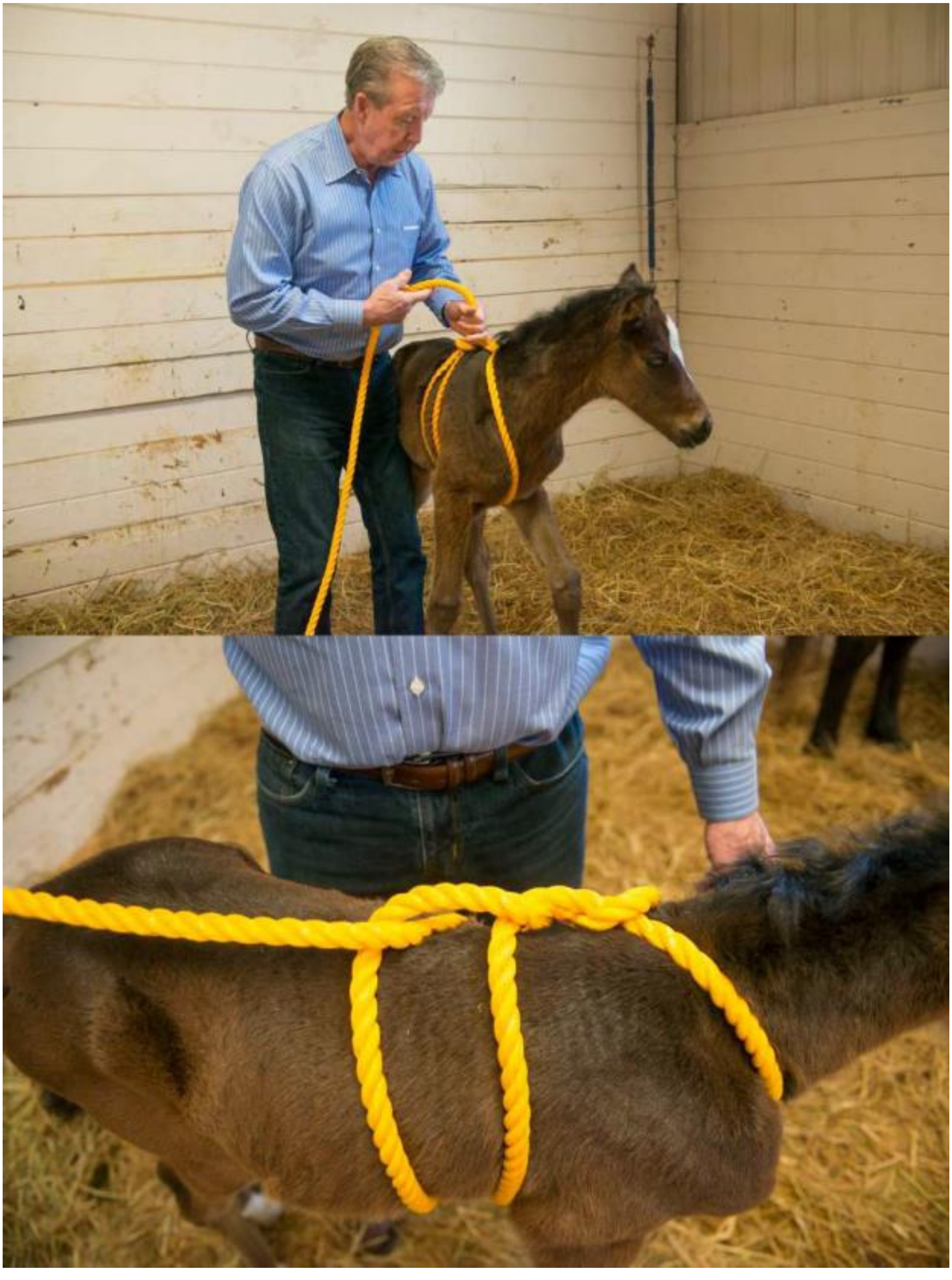

Step 5: Stand behind foal and begin to put tension on rope. The foal will then lie down.

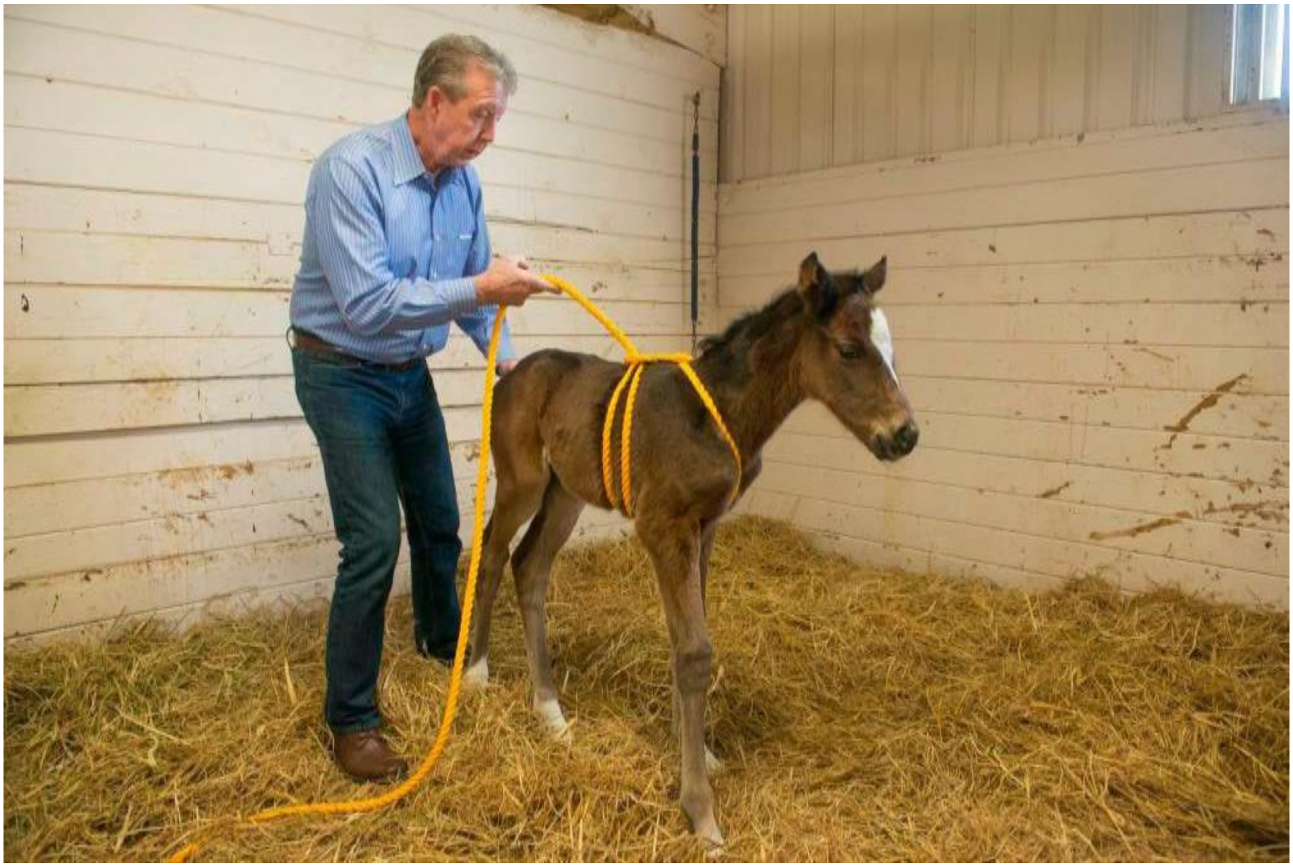

Step 6: When the foal is lying down, keep tension on rope for the next 20 minutes and release.

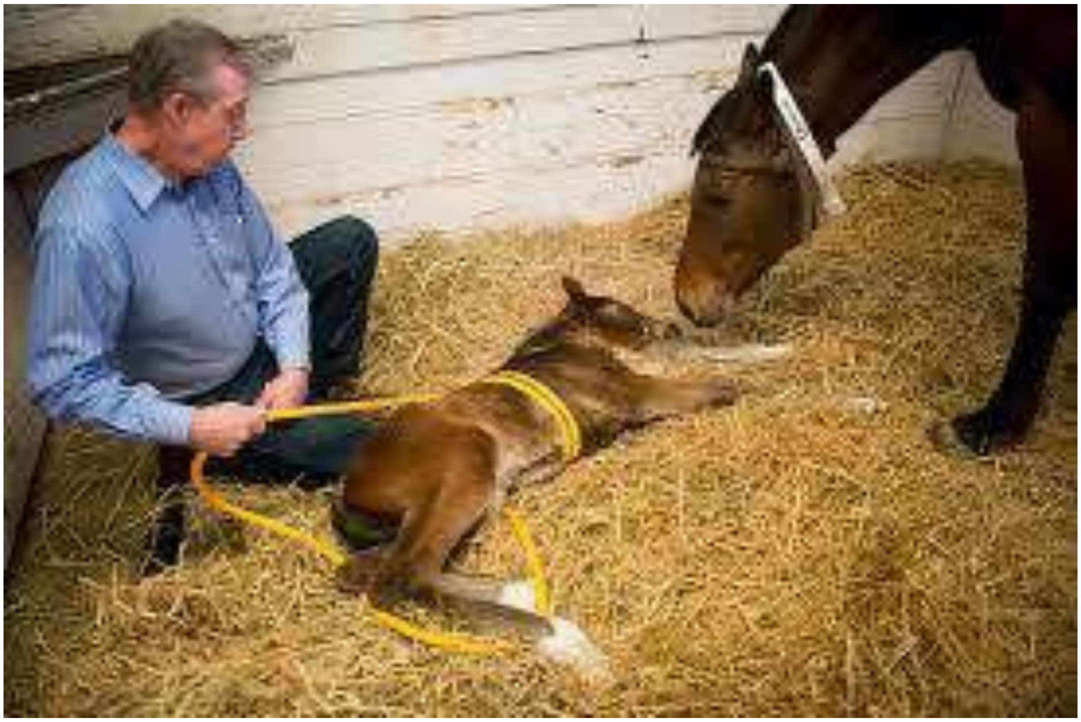

Step 6 cont'd: Luggage scale with rope attached showing suggested 10--- 20 lbs pressure

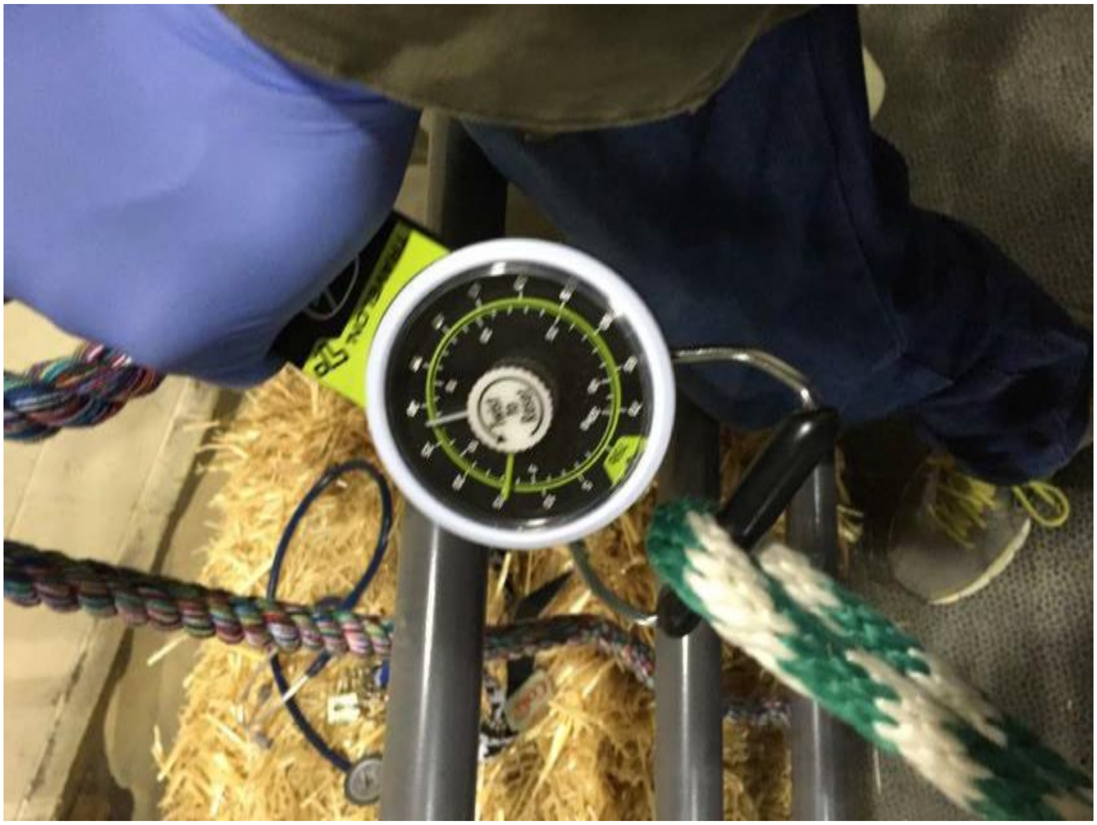

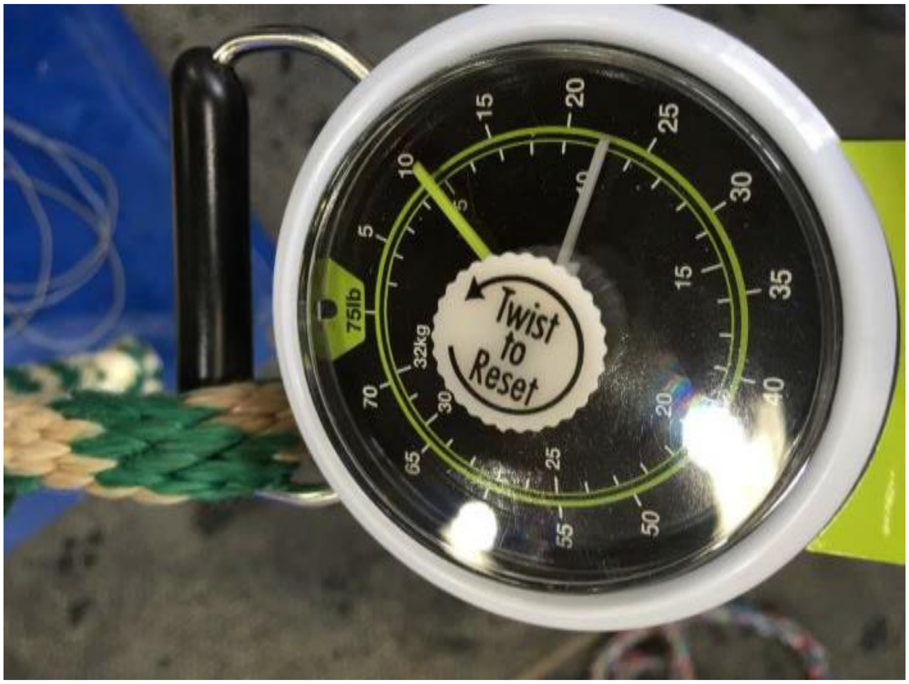

Supplement: Supplementary file 1 [file animals-07-00069-s001.pdf]
